# Pregnant women who requested a ‘108’ ambulance in two states of India

**DOI:** 10.1136/bmjgh-2017-000704

**Published:** 2018-05-03

**Authors:** Samiksha Singh, Pat Doyle, Oona Maeve Renee Campbell, Govindaraju Venkata Ramana Rao, Gudlavalleti V S Murthy

**Affiliations:** 1 Maternal and Child Health, Indian Institute of Public Health Hyderabad, Public Health Foundation of India, Hyderabad, Andhra Pradesh, India; 2 Department of Non-communicable Disease Epidemiology, Faculty of Epidemiology and Population Health, London School of Hygiene and Tropical Medicine, London, UK; 3 Department of Infectious Disease Epidemiology, Faculty of Epidemiology and Population Health, London School of Hygiene and Tropical Medicine, London, UK; 4 Emergency Medicine Learning Center and Research, GVK-Emergency Management and Research Institute, Hyderabad, Andhra Pradesh, India; 5 Department of Clinical Research, Faculty of Infectious and Tropical Diseases, London School of Hygiene and Tropical Medicine, London, UK

**Keywords:** health services research, maternal health, obstetrics, public health, descriptive study

## Abstract

We studied the pregnant women, who requested a ‘108’ ambulance in two Indian states (Andhra Pradesh (AP) and Himachal Pradesh (HP)). We conducted a cross-sectional telephone survey to study the characteristics and outcomes of those who (1) were transported using ‘108’ ambulance, (2) were sent ‘108’ ambulance but did not use it and (3) were not assigned a ‘108’ ambulance. We conducted interviews within 24 hours of clients’ call and followed them up at 48 hours, on the 7th and 28th day. 90% of pregnant women callers in AP and only 16% in HP were from poorer socioeconomic circumstances. 22.5% of women who were not provided an ambulance in HP lived in tribal areas. A higher proportion of women who were transported using ‘108’ reported either a high-risk condition (AP, 22%; HP, 27%) or an early complication in pregnancy (AP and HP, 16%), compared with the other groups (AP, 18% and 8%; HP, 19% and 16%). In AP, women who were sent an ambulance but did not use it had higher prevalence of obstetric emergency (9.8%) compared with the other groups (ambulance used, 7.4%; not assigned, 4.1%). One-fifth of women in AP and one-seventh in HP delivered by caesarean section. One woman who called, but was not transported by ‘108’, died in AP. Ten stillbirths and 22 neonatal deaths were reported in AP and 17 and 16, respectively, in HP. Strategies are required to improve ‘108’ service for tribal areas in HP. The ‘108’ services should be improved to reduce non-use of ambulance, especially for women who report obstetric emergencies.

**Summary box**

- The ‘108’ ambulance service is the largest free transport service for the pregnant women in India and is expected to provide basic obstetric and life support during the journey.
- Nine in 10 women called ‘108’ service for normal labour pain in the two states (Andhra Pardesh and Himachal Pradesh) included in this study, while about one-third of these women had a high-risk condition or early complication in pregnancy.
- A higher proportion of women who were transported using the ‘108’ ambulance reported a high-risk condition or early complication in pregnancy, compared with those who used other means of transport.
- Whereas, a higher proportion of women who called ‘108’ but used other means of transport reported an obstetric emergency.
- The use of ‘108’ service should be improved for the women with obstetric emergencies.

## Introduction

India has an estimated 45 000 maternal deaths annually, which is the second highest number globally.^1^ The maternal mortality ratio is estimated to be 167/100 000 live births.^2^ The Indian government promotes institutional delivery through a three-tier primary healthcare system.^3–5^ The transportation of referred pregnant women to appropriate health facilities and en route stabilising care plays a pivotal role in preventing maternal deaths in low-income and middle-income countries.^6–8^

In India, several innovative emergency referral transportation services are provided through the public system or public–private partnerships.^9–11^ The ‘108’ ambulance service is the largest, operating across 22 states and union territories to provide free emergency medical service for all type of emergencies and for transport of pregnant women. The service provides en route basic life support and obstetric care through trained emergency medical technicians (EMT).^9^ The service transports client preferably to the nearest government facility—the decision depends on the condition of the pregnant woman, the choice of the family or, if referred, the suggestions of referring health staff.^9^

We conducted a telephone survey in 2016, to (1) describe the demographic and clinical characteristics and outcomes of pregnant women and (2) the type of health facility used by the pregnant women who called ‘108’ service to request for an ambulance in two Indian states—Andhra Pradesh (AP) from south and Himachal Pradesh (HP) from north. The ‘108’ call centre categorised these women into three groups by use of ‘108’ ambulance, those (1) who called ‘108’ and were transported using ‘108’ ambulance, (2) who called ‘108’ and were sent an ambulance, but did not use it and (3) who called ‘108’ but were not assigned an ambulance (mostly due to non-availability of a free ambulance). Preliminary analysis of 2013–2014 data showed that ‘108’ service received 294 695 pregnancy-related calls in AP and 25 016 in HP.^12^ Of these, 90%, 8% and 2% in AP and 98%, 2% and 0.2% in HP fell into the above-stated groups, respectively. Less than 1 in 10 women in group 1 who were transported using ‘108’ ambulance had an obstetric emergency. Groups 2 and 3 were not transported by ‘108’ and may have a different proportion of obstetric complications compared with those transported.

**Figure 1:**
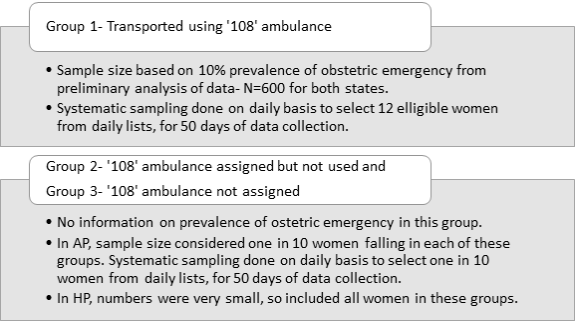
Sample size estimation and sampling strategy for study groups in each state.

We conducted two cross-sectional studies, one for each state, and computed sample size and conducted sampling as described in figure 1. For 10% prevalence of obstetric complications in women who used ‘108’ ambulance, an absolute precision of 2.5% and 80% power, we estimated a sample size of 600 transported women from each state. Data on the proportion of obstetric complications were not available for those not transported by ‘108’ ambulance. Thus, in AP, we sampled 1 in 10 women each from those who did not use an ambulance and those not assigned an ambulance. In HP, we included all the non-transported women as the numbers were very small. From the ‘108’ call centre database, we obtained the lists from all the three groups for the previous 24 hours on a daily basis for around 2 months. We conducted systematic random sampling to recruit the required number of women, every day. We scheduled the days of data collection, systematically, in a manner that each day of the week was represented. We maintained a log of all the calls, with the reason for exclusion. Follow-up calls were made at 48 hours, 7 days and 28 days after the initial call to ‘108’. We conducted interviews with the pregnant women or her husband/relative/friend, who called ‘108’ for an ambulance, in the local languages. As this was a telephone survey, only verbal consent was taken before conducting the interviews. We asked the reason for non-participation from those who did not give consent.

In both the states, more calls than expected had to be made to get the desired number of completed interviews. The online Supplementary additional file 1 describes the response to calls made and the reasons for exclusion. We finally interviewed, in AP, 582 women who were transported using ‘108’ ambulances, 215 who were sent an ambulance but did not use it and 74 who were not assigned an ambulance. From HP, we interviewed 615 women who were transported using ‘108’ and 111 women who were not assigned an ambulance. Only nine women in HP did not use an ambulance and these were not included in the analysis. In HP, due to long distances and unavailability of alternate public transport, women waited for the ambulance if assigned.

10.1136/bmjgh-2017-000704.supp1 Supplementary file 1

During interviews, for demographic characteristics, we asked for social caste (general, other backward, scheduled tribe and scheduled caste), economic class (below or above poverty line), area (rural or urban), education and occupation of both women and their husbands. The demographic stratification is same as used in the Census of India. The obstetric states—high-risk conditions, early complications in pregnancy, and obstetric emergencies—were self-reported by the user, based on known diagnoses or general awareness or perception. A ‘high-risk’ pregnancy is defined as a pregnancy with a demographic characteristic, obstetric history, or medical history that suggests an elevated risk of developing a complication.^13 14^ An ‘early complication in pregnancy’ is defined as a medical complication that developed as a consequence of pregnancy or a medical condition complicated by pregnancy during antenatal period.^14 15^ For this study, any complication in pregnancy or childbirth or within 42 days after childbirth, reported as present at the time of the call to ‘108’, and for which the call was made, was defined as an ‘obstetric emergency’. We also enquired the type of destination hospital during the first call, when most of them were still in the hospital.

Analysis of data was done using Stata V.13.0. Cumulative proportions were computed for pregnancy outcomes and mortality in the period between the call made and follow-up, for each of the study ‘108’ transport groups, separately by state. Χ^2^ test of significance for the difference between proportions was applied to compare key variables in the three study groups.

## Social, demographic and geographic characteristics

The mean age of the women in AP was 23.6 (95% CI 21.3 to 25.9) years and in HP this was 24.9 (95% CI 22.8 to 27.0) (online Supplementary additional file 2). More than 90% of women included in the survey were between 20 and 34 years of age and the majority were Hindus and from rural areas. In AP, those who accessed ‘108’ services were mostly from socially disadvantaged castes, while in HP these were mostly from general castes. HP has difficult hilly terrain, with long distances to health centres, thus even affluent people prefer using ‘108’ ambulance service as they are assured stabilising care on the route. In AP, nearly all (90%) women belonged to below-the-poverty-line strata while in HP these made up only 16% of the interviewees. About two-fifths of the pregnant women and their husbands were illiterate in AP compared with only 5% in HP. Findings from analysis of data from ‘108’ call centre logs of six states,^12^ and other studies about women who use publically financed transportation schemes (‘108’/‘102’/Janani Express Yojana),^16–20^ also show that, overall, most ‘108’ users belonged to the disadvantaged social castes, below-the-poverty-line strata and rural areas.

10.1136/bmjgh-2017-000704.supp2 Supplementary file 2

There were no large differences in sociodemographic characteristics between the study groups within each of the states. One exception was in HP, where a higher proportion of women who were not assigned an ambulance were from tribal areas (22.5%) compared with those transported using ‘108’ (4.2%) (p<0.001). Several tribal areas in HP are not connected with roads or are disconnected during heavy snow and rains; thus, ambulance may not reach them.

## High risk and early complication in pregnancy and obstetric emergency

In this study, we found that the vast majority (90%) of women in AP and HP called because of normal labour pains (table 1). Analysis of ‘108’ call centre data for the period 2013–2014 also found that between 2.7% and 9.3% of women transported by ‘108’ had an obstetric emergency, across six states.^12^ In our survey, we further enquired about demographic and obstetric high risk and complications at any time in the current pregnancy (table 2). In AP, a high-risk pregnancy was reported by 22.2% of women who were transported compared with 17.7% of those who did not use an ambulance and 18.9% of those not assigned an ambulance (p=0.350). In HP, 27% of women who were transported compared with 18.9% of women who were not assigned an ambulance had a high-risk condition in pregnancy (p=0.073). In AP, an early complication in pregnancy was reported twice as frequently by the women who were transported (16.2%) compared with other two groups (8.4% and 8.1%) (p=0.006). In HP, an early complication in pregnancy was reported by around 15% of women in both the groups.

**Table 1:** Details of the calls made to ‘108’ service for pregnant women

Use of ambulance	Andhra Pradesh			Himachal Pradesh	
	Transported using ambulance, n=582, %	Ambulance assigned but not used, n=215, %	Ambulance not assigned, n=74, %	Transported using ambulance, n=615, %	Ambulance not assigned, n=111, %
Relationship of the caller with the pregnant women					
Husband	40.0	34.4	36.5	47.2	37.8
Parents/parents in-law	19.5	17.7	19.0	16.3	19.8
Siblings/siblings in-law	21.6	26.5	21.7	16.5	19.8
Other relative/friend	6.7	10.7	8.1	8.5	14.4
Community health worker/staff at health centre	14.8	10.2	13.5	11.2	8.1
Others	0.3	0.5	1.4	0.5	0.0
Person who suggested to call ‘108’ for ambulance					
Self/relatives	84.3	88.4	76.7	84.8	94.6
Referred	15.2	10.6	20.6	15.1	5.4
Referred by community health worker	10.5	6.5	15.1	15.1	5.4
Referred by staff at health centre	4.7	4.2	5.5	0.0	0.0
Others	0.8	0.9	2.7	0.2	0.0
Reason for calling ‘108’					
Routine antenatal check-up	2.6	1.9	0.0	0.0	0.0
Antenatal with complication	4.2	2.3	0.0	0.2	0.0
Normal labour pains	89.8	88.4	95.9	90.7	94.6
Labour with complication	3.2	4.7	2.7	9.1	5.4
Postdelivery with complication	0.0	2.8	1.4	0.0	0.0
Routine postdelivery check-up	0.3	0.0	0.0	0.0	0.0
Obstetric emergency at the time of call to ‘108’	7.4*	9.8	4.1	9.3**	5.4***

Additional 7*, 30** and 2*** answered yes when asked a separate question for the presence of any complication at the time of call and which was a reason for call to ‘108’.

**Table 2:** Obstetric details and outcomes among pregnant women who called ‘108’

Use of ambulance	Andhra Pradesh			Himachal Pradesh	
	Transported using ambulance, n=582, %	Ambulance assigned but not used, n=215, %	Ambulance not assigned, n=74, %	Transported using ambulance, n=615, %	Ambulance not assigned, n=111, %
High risk in current pregnancy	22.2	17.7	18.9	27.0	18.9
Early complication in current pregnancy	16.2	8.4	8.1	15.9	16.0
Obstetric emergency at time of call to ‘108’	7.4	9.8	4.1	9.3	5.4
Any high risk/complication/emergency	36.4	30.2	24.3	41.0	35.1
Not transported to any hospital (delivered at home or stabilised)	0.0	43.7	12.2	0.0	0.9
Type of hospital pregnant woman was taken to (% of transported)					
Government primary health centre	26.5	29.8	24.6	3.1	0.9
Government community health centre	15.8	10.7	20.0	10.7	3.6
Government subdistrict/district hospital	45.5	28.1	40.0	85.2	92.7
Private clinic/hospital	12.2	31.4	15.4	1.0	2.7
Mode of delivery					
Did not deliver within the period of follow-up	7.0	6.0	2.7	8.3	9.9
Abortion	0.0	0.0	0	0.7	0
Delivered	92.3	93.9	97.3	88.3	90.1
Lost to follow-up	0.5	0.1	0.0	2.8	0
Caesarean rate (% of all delivered)	23.1	13.8	19.4	13.3	14.0
Death of the pregnant women during transfer or within 28 days of transfer	0.0	0.8	0.0	0.0	0.0
Intrauterine death/child born dead (% of all delivered)	1.7	0.5	0.0	2.8	2.0
Death of the newborn within 28 days (% of all live births)	2.7	3.0	2.8	2.1	5.1

In AP, an obstetric emergency was reported by 7.4% of women who were transported by a ‘108’ ambulance, 9.8% of the women who did not use the ambulance and 4.1% who were not assigned an ambulance (p=0.252). In contrast, a higher proportion of women in HP who were transported reported an obstetric emergency (9.3%) compared with the other group (5.4%), although this difference was not statistically significant (p=0.183). These observations suggest good birth preparedness, with elective decisions to use ‘108’ ambulances for childbirth, but reliance on more promptly available vehicles in cases of emergency. In another study from AP, the women who perceived an emergency did not wait long for an ambulance and were more likely to use any transport which was more promptly available.^18^

Overall, in both the states, the proportion of women who had a high-risk in pregnancy, early complication in pregnancy or obstetric emergency (combined) at the time of call was higher among women who were transported (36.4% in AP and 41% in HP) who did not use an ambulance (30.2% in AP) and those not assigned an ambulance (24.3% in AP and 35.1% in HP) (table 2). The evidence for differences in these proportions was strong in AP (p=0.050) and weak in HP (p=0.248).

The types of high-risk, early complication and obstetric emergency that were reported are described in the online Supplementary additional file 3. Most common high risks reported were the previous caesarean section, age less than 20 years and short stature. Women from AP also reported higher proportions of multigravida and history of neonatal death. In the current pregnancies, about one-tenth of women reported severe anaemia and 2% had high blood pressure. The most common emergencies in pregnancy were bleeding, high fever and ectopic pregnancy. Most common obstetric emergencies reported at the time of delivery were preterm labour, moderate to severe anaemia, excessive bleeding and high blood pressure.

10.1136/bmjgh-2017-000704.supp3 Supplementary file 3

Similar to this study, the Indian District Level Household Survey-4 found that about 40% of women from AP and from HP reported a complication of some kind during pregnancy.^21–23^ These women may require stabilising care and, the ‘108’ ambulance can play a critical role in managing prehospital care. In another study, it was found that EMTs in ‘108’ ambulances assisted imminent childbirth, managed the third stage of labour as well as severe pre-eclampsia and eclampsia cases en route.^24^ However, none of the EMTs administered oxytocin for PPH nor did they give magnesium sulfate to any of the pre-eclampsia and eclampsia case.^24^

## Destination hospital

Table 2 describes the pattern of use of health facilities by those who called ‘108’. In AP, 44% of women who did not use an ambulance and 12% who were not assigned an ambulance were not transferred to any health facility. Most of these women delivered or stabilised before any transport was used. The remaining were transported by other means—90% hired an auto rickshaw or a taxi in both states and, only six women in AP used another form of an ambulance.

In AP, among the women who were transported using ‘108’, almost half went to government subdistrict/district hospitals and one-quarter to primary health centres (PHCs) and community health centres (CHCs). Among the women who were transported by means other than ‘108’, use of government facilities was low and a higher proportion went to private hospitals compared with those transported by ‘108’ ambulance (p<0.001). A similar phenomenon of bypassing lower levels of care has been documented in other studies.^25 26^ In HP, more than 90% were transferred to government subdistrict/district hospitals across both the groups (p=0.009). But the use of PHCs and CHCs was less likely in HP compared with AP (table 2). In HP, the vast majority of women went to government subdistrict or district hospitals, most likely reflecting the poor availability of obstetric services at low-level facilities.^27^

In AP, among those transported using ‘108’, more than half of the pregnant women who had a high risk or complication or emergency went to higher level government hospitals, whereas half of the women who did not have any problems went to PHCs and CHCs (table 3). A higher proportion of pregnant women with an emergency (23.3%) used private facilities compared with those with high risk (17.1%) or early complication (12.8%) or none (10.0%) (table 3). The pregnant women trusted care in private sector more than public. Such diversion of obstetric emergencies for care in private sector would add considerably to out of pocket expenditure compared with the care in public hospitals. In HP, the type of destination hospital did not vary with the type of complications and use of ‘108’—private obstetric care was less available in remote areas.

**Table 3:** Type of obstetric complication in pregnant women who called ‘108’ with type of destination hospital in study states

	Transported using ‘108’ ambulance					Not transported by ‘108’ ambulance				
	High risk in current pregnancy	Complication in current pregnancy	Any emergency at time of call	Any high risk/complication/emergency	None	High risk in current pregnancy	Complication in current pregnancy	Any emergency at time of call	Any high risk /complication /emergency	None
**Andhra Pradesh, %**	**n=129**	**n=94**	**n=43**	**n=212**	**n=370**	**n=52**	**n=24**	**n=24**	**n=83**	**n=206**
Primary health centre	15.5	20.2	20.9	19.3	30.5	22.6	35.0	25.0	27.1	28.3
Community health centre	10.9	12.8	7.0	11.8	18.1	6.5	15.0	0.0	6.8	17.3
Government subdistrict/district hospital	56.6	54.3	48.8	52.8	41.4	32.3	35.0	45.0	35.6	30.7
Private clinic/hospital	17.1	12.8	23.3	16.0	10.0	38.7	15.0	40.0	30.5	23.6
**Subtotal transported**	**100.0**	**100.0**	**100.0**	**100.0**	**100.0**	**100.0**	**100.0**	**100.0**	**100.0**	**100.0**
Not transported	0.0	0.0	0.0	0.0	0	40.4	16.7	8.3	28.9	38.3
**Himachal Pradesh, %**	**n=166**	**n=96**	**n=57**	**n=252**	**n=363**	**n=21**	**n=17**	**n=6**	**n=39**	**n=72**
Primary health centre	3.0	4.2	1.8	3.2	3.0	0	0	0	0	1
Community health centre	9.0	6.3	12.3	9.5	11.6	0	0	16.7	2.6	4.2
Government Sub-District/District hospital	86.7	89.6	84.2	86.1	84.6	95.2	94.1	83.3	92.3	91.7
Private clinic/hospital	1.2	0.0	1.8	1.2	0.8	4.8	5.9	0	5.1	1.4
Not transported	0.0	0.0	0.0	0.0	0.0	0	0	0	0	1.4

## Outcomes of pregnancy

Table 2 and figures in the online Supplementary additional file 4 describe the mode of delivery, outcome of pregnancy, place of outcome and follow-up for all the study groups. For the description of results in this section and the next, the group of women in AP who did not use a ‘108’ ambulance despite of being sent one and those who were not assigned an ambulance were combined to form a group not transported by ‘108’, while in HP this was the only group not assigned an ambulance.

10.1136/bmjgh-2017-000704.supp4 Supplementary file 4

Between 5.2% and 7.0% of women in AP and 8.3% and 9.9% in HP did not deliver within 28 days following the call to ‘108’ (table 2). No women in AP and only four women in HP had abortions (type not known). Of the women who delivered in AP, 0.9% transported by ‘108’ and 30.3% not transported by ‘108’ delivered at home (data not shown). In HP, these proportions were 5.7% and 1.0%, respectively. In AP, a high proportion of women delivered by caesarean section—23.1% of those transported by ‘108’ and 15.3% of those not transported by ‘108’ (p=0.015). In HP, caesarean section rates were lower—13.2% among those transported by ‘108’ and 14% of those not transported by ‘108’ (p=0.842). These proportions are higher than those reported in another study on the ‘108’ service (8%).^24^ The latter study found that the caesarean rates were more likely if the women were from a rural area, was first taken to a PHC, had history of caesarean section or were nulliparous (p<0.05).^24^

Only one pregnant woman, who was not transported by ‘108’, died in AP, and none in HP. Ten stillbirths and 22 neonatal deaths were reported in AP and 17 and 16, respectively, in HP. The proportions of neonatal deaths were similar across the transport groups in AP (between 2.7% and 3.0% of all live births) but in HP, neonatal deaths were more than twice as common in women not transported by ‘108’ (5.1% of all live births) compared with those transported by ‘108’ (2.1% of all live births) (table 2). However, there were no statistical differences in the proportions of stillbirth and neonatal deaths between transported and non-transported women in either of the two states (p>0.05). Stillbirth rates recorded in our study were more than twice likely compared with the population rates found in SRS 2012–2013 surveys (stillbirth rate in AP—0.5% and HP—1.2%).^2^ This might be explained by the high proportion of pregnant users of the ‘108’ service who had a high-risk condition or early complication. These women are more likely to have adverse pregnancy outcomes. The neonatal mortality rate in the offspring of ‘108’ users in our study was similar to findings from SRS 2012–13 (NMR in AP—26 and HP—25 per 1000 live birth).^2^

The findings of this study are generalisable only to women who called ‘108’. A larger comparison of group of women who called ‘108’ and did not use it and an additional group of women who did not call ‘108’ at all, will be required to be able to generalise these findings to the general population. A large proportion of phones of callers of pregnant women could not be reached, which could lead to selection bias. The diagnosis was self-reported by the client, and these may not correspond to the true clinical picture and can be over or under-estimated. However, women’s own perceptions of high-risk conditions, complications and emergencies in pregnancy, and the ready availability of other means of transport, may contribute to the decision to call for a ‘108’ ambulance and its use.^16^ Several large population-based surveys in India use client’s view of their health status.^21–23^ The sample sizes could not be estimated for the groups not transported by ‘108’ ambulance, and their sample sizes were also small. Thus, we restricted the statistical comparisons only for key obstetric conditions, destination hospitals and key outcomes.

## Conclusion

A higher proportion of women who used a ‘108’ ambulance in AP were from poor socioeconomic circumstances, while in HP, the users were mostly from the general class. In HP, tribal women were less likely to receive an ambulance. Women transported using a ‘108’ ambulance were more likely to have high-risk conditions and early complications and use government facilities, while women transported using other means were more likely to have an obstetric emergency and use private facilities. There were no large differences in adverse pregnancy outcomes among those transported using ‘108’ ambulance than those not transported; however, larger studies are required to make valid conclusions. The findings suggest that the ‘108’ service should adopt strategies to reach the poor and unreachable in HP. Strategies are required to improve the use of ‘108’ services for women who report obstetric emergencies.
