# Supplementary material for: Pregnant women who requested a ‘108’ ambulance in two states of India
Source: BMJ Glob Health. 2018 May 3;3(3):e000704. doi: 10.1136/bmjgh-2017-000704 (PMC5935162; doi:10.1136/bmjgh-2017-000704)
Supplement: Supplementary file 1 [file bmjgh-2017-000704supp001.pdf]

Additional file 1: Response to calls made to pregnant women/relatives/others who called '108' service

	Total calls made	Caller not reachable	Unable to provide information	Reverse transport	Eligible calls (Sub-total)	Consent not given, n(% of eligible)	Complete interview, n(% of eligible)
**Andhra Pradesh**							
Transported using ambulance, N(%)	**1207**	208 (17.2)	185 (15.3)	146 (12.1)	**668** (55.3)	86 (12.9)	582 (87.1)
Ambulance assigned but not used, N(%)	**570**	126 (22.1)	91 (16.0)	80 (14.0)	**273** (47.9)	58 (21.2)	215 (78.8)
Ambulance not assigned, N(%)	**186**	50 (26.9)	21 (11.3)	17 (9.1)	**98** (52.7)	24 (24.5)	74 (75.5)
**Total, N(%)**	**1963**	**384 (19.6)**	**297 (15.1)**	**243 (12.4)**	**1026 (52.3)**	**168 (16.4)**	**858 (83.6)**
**Himachal Pradesh**							
Transported using ambulance, N(%)	**899**	119 (13.2)	98 (10.9)	0	**682** (75.9)	67 (9.8)	615 (90.2)
Ambulance not assigned, N(%)	**170**	23 (13.5)	21 (12.4)	0	**126** (74.1)	15 (11.9)	111 (88.1)
**Total, N(%)**	**1069**	**142 (13.3)**	**119 (11.1)**	**0**	**808 (75.6)**	**82 (10.1)**	**726 (89.9)**

Caller not reachable = Phone number was not reachable or was unanswered even after calling five times over a span of two hours. **Unable to provide information** = Caller who could not provide information about the pregnant women were not included. **Reverse transport** = Mother and child were transported back from hospital to home and were not eligible. **Consent not given** caller refused to participate in the survey.
