# Supplementary material for: Pregnant women who requested a ‘108’ ambulance in two states of India
Source: BMJ Glob Health. 2018 May 3;3(3):e000704. doi: 10.1136/bmjgh-2017-000704 (PMC5935162; doi:10.1136/bmjgh-2017-000704)
Supplement: Supplementary file 2 [file bmjgh-2017-000704supp002.pdf]

Additional file 2: Socio-demographic characteristics of pregnant women who called '108' in the study states

	Andhra Pradesh			Himachal Pradesh	
**Use of ambulance**	**Transported using ambulance N= 582, %**	**Ambulance assigned but not used N= 215, %**	**Ambulance not assigned N= 74, %**	**Transported using ambulance N= 615, %**	**Ambulance not assigned N= 111, %**
**Age**					
< 20 years	7.0	6.0	5.4	3.1	8.1
20-24 years	64.1	58.6	66.2	48.1	45.9
25-29 years	23.7	25.6	21.6	36.4	36.0
30-34 years	3.8	7.0	2.7	9.4	6.3
≥ 35 years	0.3	1.9	0.0	2.9	3.6
Don't know	0.5	0.9	4.1	0.0	0.0
**Social caste**					
Other backward caste	51.4	56.7	55.4	13.8	14.4
Scheduled caste	33.8	25.1	24.3	25.7	27.0
Scheduled tribe	13.9	14.9	13.5	5.7	6.3
General	0.7	2.3	6.8	31.9	30.6
Don't know	0.2	0.9	0.0	22.9	21.6
**Economic class**					
Below poverty line	93.6	90.2	93.2	15.9	18.9
Above poverty line	0.9	1.4	0.0	49.3	45.0
Don't know	5.5	8.4	6.8	34.8	36.0
**Pregnant women worked for earnings during this pregnancy**	45.2	47.4	42.3	5.4	8.1
**Education status of pregnant women**					
Illiterate	38.0	43.7	37.8	9.1	8.1
Primary school	12.2	14.4	20.3	7.5	12.6
Middle school	29.0	21.4	24.3	39.7	42.3
12 grade	9.8	9.3	6.8	27.5	27.9
Graduate or above	6.5	4.2	5.5	11.5	7.2
Don't know	4.5	7.0	5.4	4.8	1.8
**Education status of husband of pregnant women**					
Illiterate	39.0	43.3	43.2	4.6	6.3
Primary school	11.3	12.6	10.8	7.2	7.2
Middle school	23.2	23.2	28.4	43.4	47.7
12th	5.8	4.2	1.4	29.6	26.1
Graduate or above	12.7	7.5	9.5	9.9	11.7
Don't know	7.9	9.3	6.8	5.4	0.9
**Religion**					
Hindu	69.1	72.1	70.3	95.3	99.1
Muslim	0.9	2.3	6.8	4.1	0.9
Others	29.9	24.2	23.0	0.5	0.0
Don't know	0.2	1.4	0.0	0.2	0.0
**Area**					
Rural	84.5	87.4	85.1	94.5	75.7
Tribal	6.5	5.1	2.7	4.2	22.5
Urban	8.9	7.4	12.2	1.3	1.8

Chi-square test of proportions used for comparisons between groups, within each of the states. All comparisons had p values < 0.05, except the variable Area in HP where p value was less that <0.001.
