# Supplementary material for: Pregnant women who requested a ‘108’ ambulance in two states of India
Source: BMJ Glob Health. 2018 May 3;3(3):e000704. doi: 10.1136/bmjgh-2017-000704 (PMC5935162; doi:10.1136/bmjgh-2017-000704)
Supplement: Supplementary file 3 [file bmjgh-2017-000704supp003.pdf]

Additional file 3: Details on high-risk pregnancy, early complication in pregnancy and obstetric emergency

	Andhra Pradesh			Himachal Pradesh	
Use of ambulance	Transported using ambulance N= 582, %	Ambulance assigned but not used N= 215, %	Ambulance not assigned N= 74, %	Transported using ambulance N= 615, %	Ambulance not assigned N= 111, %
**High risk in current Pregnancy**					
Age < 20	7.0	6.0	5.4	3.1	8.1
Primigravida >35	0.1	0.0	0.0	0.9	2.5
Height < 150 cm	5.2	2.8	1.4	13.5	7.2
≥ 4 pregnancies	3.3	3.3	5.4	0.02	0.0
History of medical illness	0.0	0.0	0.0	3.6	2.8
History of complications in previous pregnancy/ pregnancies (women with gravida 2 or more)					
	**N=315, %**	**N=130, %**	**N=42, %**	**N=287, %**	**N=35, %**
Birth by operation over abdomen	16.5	7.1	9.5	13.6	6.5
Obstructed labour	0.7	1.6	0.0	0.0	0.0
High blood pressure	0.0	0.8	0.0	0.0	0.0
Convulsions/ fits	0.0	0.0	0.0	0.0	0.0
High sugar	0.3	0.0	0.0	0.7	0.0
Neonatal death	2.3	3.9	4.8	0.0	0.0
3 or more consecutive abortions/ still births	0.3	0.0	0.0	0.3	0.0
Premature births	1.7	0.0	0.0	0.0	0.0
Multiple pregnancies	0.0	0.0	0.0	0.3	0.0
	**N= 582, %**	**N= 215, %**	**N= 74, %**	**N= 615, %**	**N= 111, %**
**Any high risk in current pregnancy**	**22.2**	**17.7**	**18.9**	**27.0**	**18.9**
**Early complication in pregnancy or post-delivery**					
**History of complication in current pregnancy**					
Haemoglobin < 7 gm%	13.2	6.5	6.8	9.8	11.7
High BP	2.1	2.8	1.4	2.4	0.9
Convulsions/ fits	0.3	0.0	0.0	0.0	0.9
High sugar	0.3	0.0	0.0	0.5	0.0
Bleeding from vagina	0.9	0.0	0.0	1.0	0.9
High fever	0.5	0.0	0.0	4.1	1.8
Foul smelling discharge from vagina	0.3	0.0	0.0	0.5	0.9
Others	0.2	0.0	0.0	1.8	0.0
**Any early complication in current pregnancy**	**16.2**	**8.4**	**8.1**	**15.9**	**16.0**
**Emergency in pregnancy or delivery or post-delivery**					
**Obstetric emergency in pregnancy**					
Bleeding in pregnancy	1.0	0.9	0.0	0.0	0.0
High fever	0.7	0.7	0.0	0.3	0.0
Ectopic pregnancy/rupture	0.7	0.9	0.0	0.0	0.0
High BP/ convulsions	0.5	0.0	0.0	0.2	0.0
Foul smelling discharge	0.5	0.0	0.0	0.7	0.9
Foetal distress	0.5	0.7	0.0	0.0	0.0
Excessive vomiting	0.0	0.0	0.0	0.7	0.0
**Obstetric emergency during delivery**					
Pre-term labour	2.1	4.2	0.2	0.2	0.0
Moderate to Severe anaemia	1.5	0.5	0.0	0.0	0.0
Excessive bleeding	1.0	1.4	0.0	5.0	4.5
High BP/ convulsions	0.9	3.3	1.4	1.3	0.0
High fever	0.0	0.0	0.0	0.8	0.0
Multiple foetus/ Malpresentation	0.2	0.5	0.0	0.2	0.0
Others	0.7	0.9	0.0	0.2	0.0
**Any Obstetric emergency at time of call**	**7.4**	**9.8**	**4.1**	**9.3**	**5.4**
