# Supplementary material for: Pregnant women who requested a ‘108’ ambulance in two states of India
Source: BMJ Glob Health. 2018 May 3;3(3):e000704. doi: 10.1136/bmjgh-2017-000704 (PMC5935162; doi:10.1136/bmjgh-2017-000704)

Additional Figure 1: Follow up of pregnant women who were transported using '108' ambulance with their outcomes in state of Andhra Pradesh

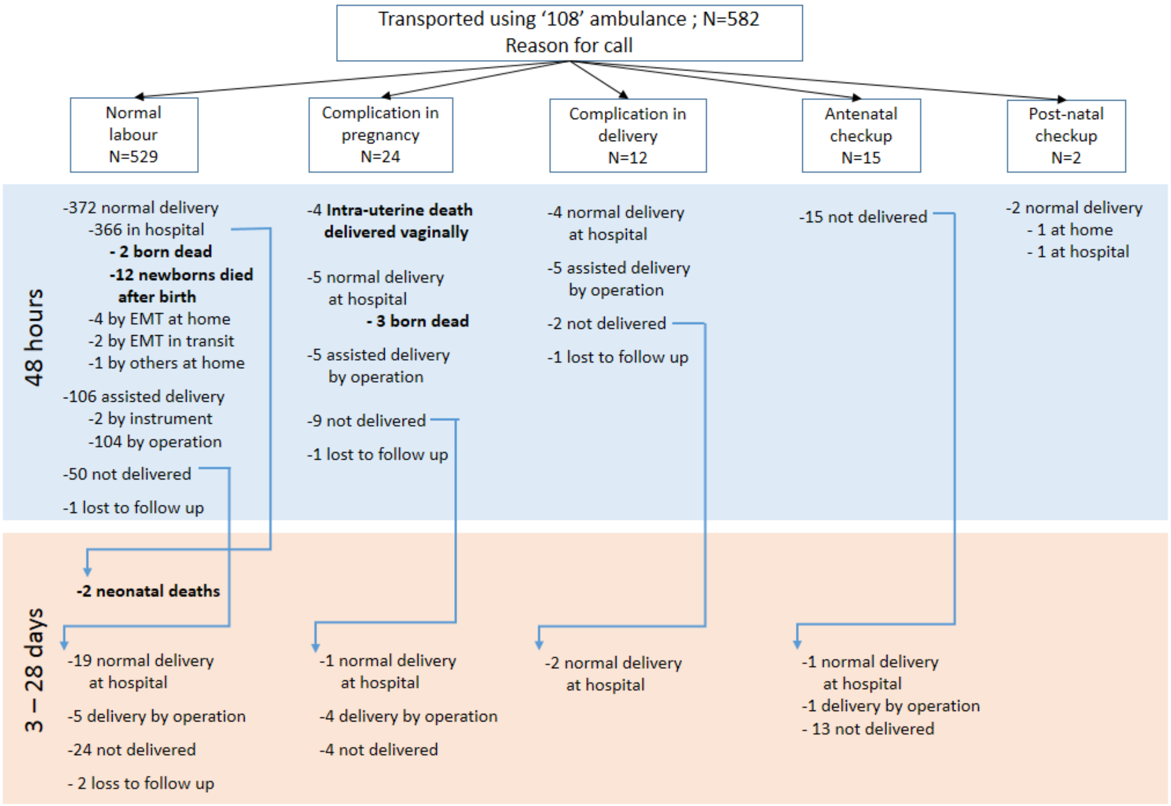

Additional Figure 2: Follow up of pregnant women who were not transported by '108' ambulance with their outcomes in state of Andhra Pradesh

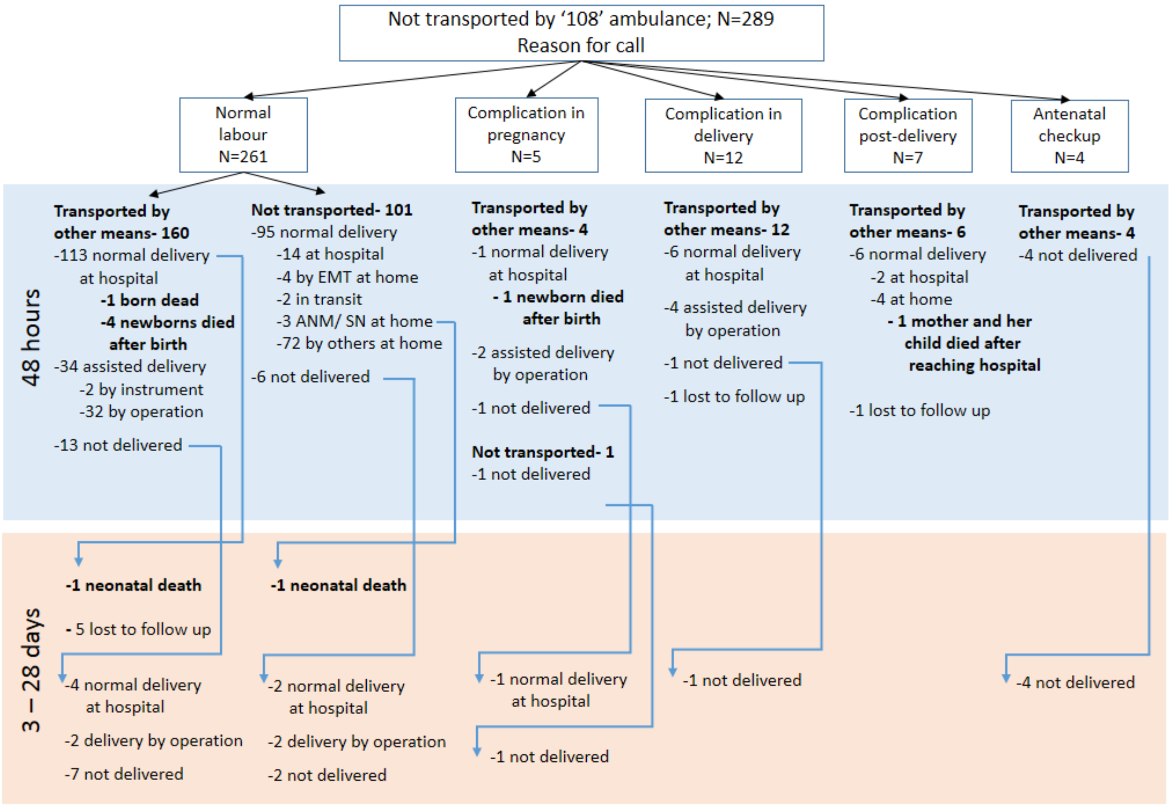

Additional Figure 3: Follow up of pregnant women who were transported using '108' ambulance and who were not transported by '108' ambulance with their outcomes in state of Himachal Pradesh

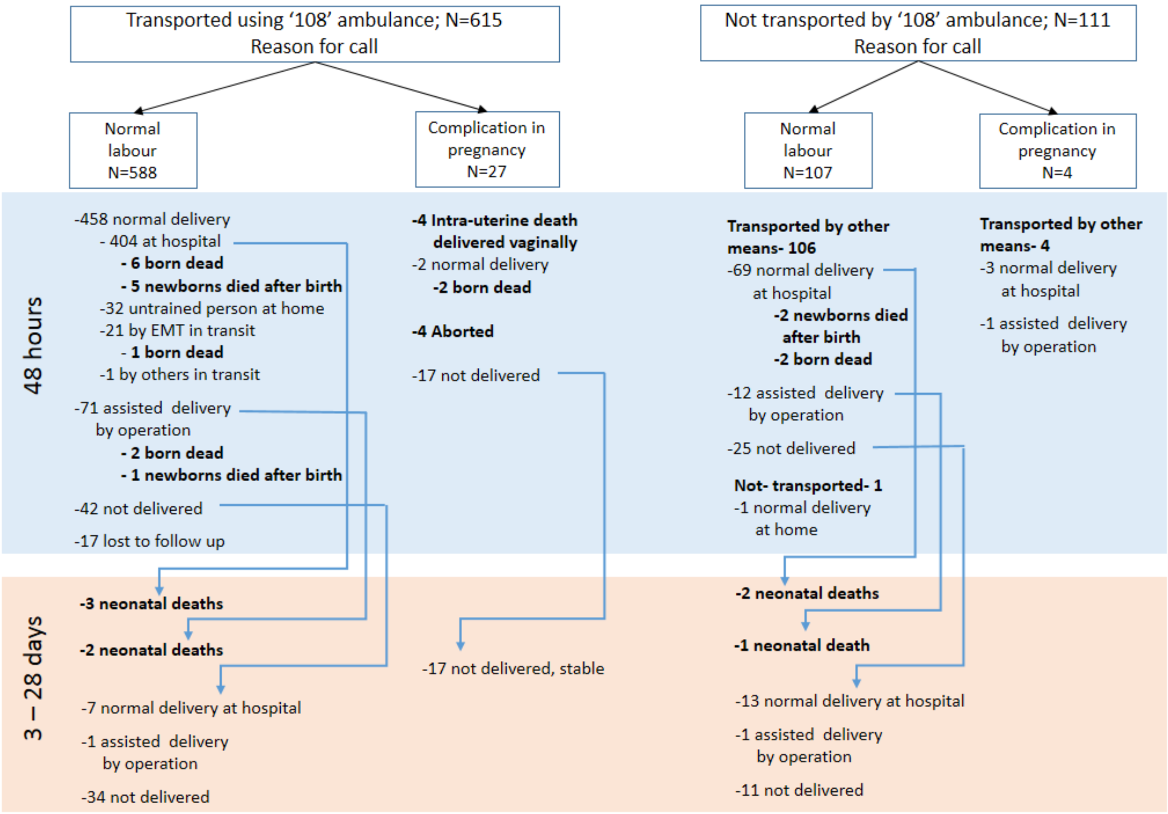

Supplement: Supplementary file 4 [file bmjgh-2017-000704supp004.pdf]
